# A Small Molecule Compound, Berberine Reduces IgE but Not IgG Production via Promoting miRNA-34a-p53 Axis

**DOI:** 10.3390/cells14221799

**Published:** 2025-11-17

**Authors:** Michelle Carnazza, Madison Spears, Raj K. Tiwari, Jan Geliebter, Nan Yang, Xiu-Min Li

**Affiliations:** 1 R&D Division, General Nutraceutical Technology LLC, Briarcliff Manor, NY 10510, USA; michelle.carnazza@gnt-us.com (M.C.); nan.yang@gnt-us.com (N.Y.); 2 Department of Pathology, Microbiology & Immunology, New York Medical College, Valhalla, NY 10595, USA; mspears@student.touro.edu (M.S.); raj_tiwari@nymc.edu (R.K.T.); jan_geliebter@nymc.edu (J.G.); 3 Department of Otolaryngology, New York Medical College, Valhalla, NY 10595, USA; 4 Department of Dermatology, New York Medical College, Valhalla, NY 10595, USA

**Keywords:** berberine, natural compound, IgE, miR-34, p53

## Abstract

Current therapeutic strategies for IgE-mediated diseases are limited. The drawbacks include adverse reactions, ineffectiveness, and relapses. Natural compound berberine (BBR) may combat this therapeutic gap through sustained transcriptional regulation of IgE. Human tonsil cells were cultured in the presence or absence of BBR to establish dose-dependent effects on IgE, IgG, and cell viability. IgE-producing plasma cells (U266, IgE plasma cells) and IgG-producing plasma cells (ARH-77, IgG plasma cells) were used as surrogate cells to validate dose-dependent effects on IgE and IgG production, respectively. At 10 μg/mL BBR, cell viability and proliferation were determined, and cells were harvested for protein, RNA, and miRNA and analyzed by Western blot and qPCR. BBR treatment of human tonsil samples resulted in reduced IgE production (*p* < 0.001) with no effect on IgG levels or cell viability. BBR demonstrated sustained, dose-dependent inhibition of IgE production by IgE plasma cells (*p* < 0.001), without affecting IgG production by IgG plasma cells. There was no significant reduction in cell viability of either cell type. Proliferation was reduced in IgE plasma cells (*p* = 0.02), but not IgG plasma cells. Assessment of IgE regulation and cell cycle at the RNA level revealed that BBR reduced IgE heavy chain expression and CCND1 (*p* < 0.01), with increased the GADD45A expression of IgE plasma cells, only (*p* = 0.016). At the protein level, BBR increased p53 (*p* = 0.02) and CDKN1C (*p* = 0.03), and decreased CDK2 (*p* = 0.01) expression of IgE plasma cells, only. Investigation of miRNAs implicated in B cell and p53 regulation demonstrated increased p53 and GADD45A activator, miR-34a (*p* = 0.04). miRNAs that are present in IgE plasma cells allow for specific effects on B cells and cell cycle genes by BBR, that are not present in IgG plasma cells. A novel mechanism for specific suppression of IgE by BBR highlights miR-34a, involved in the p53 pathway and B cell development, and may be crucial to pathological IgE production.

## 1. Introduction

The prevalence of food allergies driven by immunoglobulin (Ig) E has been rising sharply in incidence in recent decades. Approximately 20 million Americans, around 8% of children and 6% of adults, are affected, arising from a specific and reproducible immune reaction to specific foods [1]. A group of 8–9 foods accounts for nearly 90% of allergic events, with peanuts, tree nuts, fish, shellfish, and sesame being the most persistent triggers [2]. These long-lasting allergies are responsible for a majority of the severe and life-threatening anaphylactic reactions. Current clinical management in Western medicine relies mainly on strict avoidance, rescue medication, including epinephrine, corticosteroids and antihistamines, and immunotherapy. Yet, available treatments remain inadequate due to high cost, adverse reactions, variable efficacy, frequent relapses, and the paradoxical rise in IgE levels that may heighten the susceptibility for further reactions [3]. With no long-term or curative intervention established, the burden on allergy patients remains substantial.

Understanding the mechanism of IgE-mediated food allergy has highlighted the potential for natural products that inhibit pathological IgE. Naturally occurring compounds with immunoregulatory activity are gaining attention as potential preventative and treatment options. Identification of the active compounds responsible for the prevention of food allergy responses revealed berberine (BBR) as a major active ingredient of the Chinese food allergy herbal formula (FAHF)-2 [4,5]. It is a natural isoquinoline alkaloid common to the rhizome, roots, and stem barker of the *Berberis* genus of Chinese herbs. BBR is being used for the prevention and treatment of a variety of diseases due to its antioxidant, antibacterial, anticancer, and anti-inflammatory actions. BBR has also demonstrated strong inhibitory effects on IgE production [4,5,6]. However, the mechanisms underlying IgE regulation by BBR are largely unknown.

It has been reported that immunoglobulin transcription, and hence their synthesis, only takes place at certain times of the cell cycle, particularly during the late G1 and S phase for IgM and IgG [7]. The transition to the IgE isotype by class switching recombination is subject to strict regulatory control. Upon activation of IgE class switching, differential expression of over 1000 genes for biological processing terms relating to the cell cycle was demonstrated [8]. Genes overexpressed in IgE plasma cells compared to IgG plasma cells were involved in translation initiation, cell cycle phase transition, and mitotic cellular division, owing to their increased proliferative and cycling capacity [9]. This included the upregulation of negative regulators of the cell cycle in IgG plasma cells and positive regulators in IgE plasma cells including cell division cycle 25B, myc, and E2F-transcription factor 2 [9]. The induction of allergic responses may therefore be a result of the dysregulation of the cell cycle. Profiling the IgE plasma cell lineages for cell-cycle programs is underexplored.

Transcription is regulated at both the coding and non-coding level, involving proteins such as transcription factors, and non-coding RNAs (ncRNAs), respectively. Transcription factors and proteins function as activators, suppressors, co-activators, and chromatin modifiers that can either promote or silence transcription. The ncRNAs, including microRNAs (miRNAs) and long ncRNAs (lncRNAs), do not encode proteins, but scaffold transcriptional machinery or degrade regulatory transcripts. Post-transcriptional regulation of allergic responses by miRNAs has been previously implicated in asthmatic patients [10]. Modulation of IgE plasma cell transcription may be more specific due to the non-coding regulation of transcription.

Few therapies directly target IgE production at the transcriptional or plasma cell level, underscoring the potential of natural compounds to specifically regulate IgE transcription. The objective of this study was to determine the effect of BBR on tonsil cultures and surrogate IgE-producing cells (U266) compared to IgG-producing cells (ARH-77), focusing on the potential IgE-specific transcription regulation at the coding and non-coding level. We demonstrated the specificity of BBR for IgE production, over IgG production, that was characterized by alterations to the tumor protein 53 (p53) and cell cycle pathway. The specificity is crucial, as BBR may treat IgE-mediated disorders without significantly impacting the overall immune function.

## 2. Materials and Methods

### 2.1. Cell Culture and Treatment

Tonsil cells were collected after children (4–8 years) undergoing routine tonsillectomy at Westchester Medical Center consented to it, following New York Medical College Institutional Review Board approval protocol (IRB #12874). Immediately following surgical removal, the tonsils were placed in ice-cold complete Roswell Park Memorial Institute 1640 (RPMI) medium, supplemented with 1% penicillin-streptomycin, 0.5% sodium pyruvate, and 10% heat inactivated fetal bovine serum (FBS). Tissues were mechanically dissociated on ice into 3–10 mm fragments and passed through a 100 µm cell strainer in cold RPMI to generate a single-cell suspension. The resultant single cell suspension was layered onto Ficoll and centrifuged at 700× *g* for 25 min at room temperature without braking. Mononuclear cells were collected from the interphase, washed three times with cold phosphate-buffered saline, and resuspended in RPMI. Cell counts and viability were determined using trypan blue exclusion. Cells were cryopreserved in 90% FBS +10% dimethyl sulfoxide (DMSO) at ≤2 × 10^7^ cells/mL. For tonsil cell treatment, cell concentration was adjusted to 4 × 10^6^/mL in complete RPMI. Cells were stimulated with human anti-CD40 (1 µg/mL) (AB_395961, BD Biosciences, San Jose, CA, USA) and recombinant human interleukin (IL)-4 (100 ng/mL) (R&D Systems, Minneapolis, MN, USA) in combination with Toll-like receptor agonists Poly I:C and Pam3CSK4. BBR (U.S. Times Technology, Briarcliff Manor, NY, USA) was prepared to a 40 mg/mL concentration in DMSO for all experiments. For tonsil samples, BBR was prepared for a final volume of 10 μg/mL or equivalent volume of DMSO as vehicle control, and cells were incubated for 10 days in 37 °C incubator supplied with 5% CO_2_. Tonsil cells were assessed for viability and supernatants were harvested for Enzyme-linked Immunosorbent Assay (ELISA).

The IgE-producing human myeloma U266 cell line and IgG-producing human myeloma ARH-77 cell lines were obtained from American Type Culture Collection, (ATCC, Rockville, MD, USA). Cells were cultured in RPMI-1640 medium supplemented with 10% fetal bovine serum, 1% penicillin-streptomycin, and 1 mM sodium pyruvate at 37 °C under 5% CO_2_. For treatment, cells were seeded on 24-well plates at an initial concentration of 2 × 10^5^ cells/mL. Berberine at different concentrations (2.5, 5, 10 μg/mL) or DMSO at the equivalent volume (0 μg/mL) was added. After 1, 3, and 6 days, supernatants were harvested for ELISA. Additionally, cells on day 6 of treatment with 10 μg/mL were assessed for viability and harvested for RNA, protein, and miRNA isolation.

### 2.2. ELISA

Human IgE and IgG levels from tonsil or cell culture supernatants were measured using ELISA kits (Mabtech, Cincinnati, OH, USA). Briefly, cells were coated with unconjugated monoclonal antibody overnight. Plates were blocked and incubated with samples for 2 h. Following detection and addition of 3,3′,5,5′-tetramethylbenzidine substrate, Ig levels were determined by optical density at 405 nm.

### 2.3. Cell Viability and Proliferation

Cell viability and cell count were assessed by Trypan Blue Exclusion counting on a hemocytometer under brightfield microscopy. Cell proliferation was assessed with a 2,3-Bis-(2-Methoxy-4-Nitro-5-Sulfophenyl)-2H-Tetrazolium-5-Carboxanilide (XTT) assay following manufacturer’s instructions. Briefly, cells were spun down and washed with phosphate-buffered saline. A total of 1 mg XTT (Thermo Scientific, Waltham, MA, USA) and 2.5 μL N-methyl dibenzopyrazine methyl sulfate per 1 mL of clear 1X RPMI were added at 100 μL per well and after 6 h, viability was determined by optical density at 630 and 450 nm.

### 2.4. RNA Isolation and qRT-PCR Analysis

RNA was isolated using the Qiagen RNeasy Mini kit following the manufacturer’s protocol (Qiagen, Germantown, MD, USA). Briefly, cells were lysed and lysate was loaded onto the column, and total RNA was eluted. RNA concentration was determined on a Nanodrop and utilized for quantitative reverse transcriptase polymerase chain reaction (qRT-PCR) analysis. qRT-PCR was performed with PCR Biosystems 1-Step SyGreen Lo-Rox kit (PCR Biosystems, Wayne, PA, USA) according to the manufacturer’s instructions. Glyceraldehyde-3-Phosphate Dehydrogenase (GAPDH) was used as a loading control for normalization. Oligos for primer pairs were obtained through Azenta (Table S1: Primer Pairs). The QuantStudio 5 Real-Time PCR Instrument (96-Well) 0.2 mL Block (Applied Biosystems by Thermo Fisher, Waltham, MA, USA) was used for qRT-PCR and analysis.

### 2.5. Protein Isolation and Western Blot Analysis

Protein was isolated by incubation with Radioimmunoprecipitation assay (RIPA) buffer (50 mM Tris-HCl pH 7.4, 150 mM NaCl, 0.2% sodium deoxycholate, 0.1% SDS, 0.5% NP-40, and 1μM Pefebloc) and Halt protease inhibitor. Protein concentration was determined by Biorad protein assay dye reagent concentrate. Samples were loaded in sodium dodecyl sulfate polyacrylamide gel electrophoresis gels and subjected to electrophoresis and a semi-dry transfer was performed with Transblot turbo onto a polyvinylidene fluoride membrane (Biorad, Hercules, CA, USA). Membranes were blocked and probed with primary antibodies overnight at 4 °C with agitation. The membranes were washed and probed with their target secondary antibody for 2 h at room temperature and washed. Chemiluminescence substrate was added to image the bands. Following imaging, membranes were stripped in stripping buffer (Thermo Fisher Scientific, Waltham, MA, USA) for 15 min and then blocked for 1 h. Analysis was performed on ImageJ (v1.54J) and analyzed in Microsoft Excel.

### 2.6. miRNA Isolation and Analysis

RNA was isolated with Zymo Research Direct-Zol RNA Miniprep kit per the manufacturer’s instructions (Zymo Research, Irvine, CA, USA). Briefly, cells were lysed in TriZol and purified with the provided columns. miRNA was isolated and quantified with Takara Bio (Takara Bio USA, Inc., San Jose, CA, USA) Mir-X miRNA First-Strand Synthesis and TB Green qRT-PCR kits. Briefly, the RNA sample was polyadenylated and reverse transcribed, and then qPCR was performed. The miRNA-specific 5′ primer was designed with miRbase.org as recommended. The provided U6 forward and reverse primers were used as normalization controls.

### 2.7. Statistics

All experiments were performed in triplicate. Statistical analysis was performed in GraphPad prism software for significance (Version 10.0, GraphPad Software, La Jolla, CA, USA). One-way analysis of variance was performed followed by Bonferroni correction for all pairwise comparisons. For skewed data, differences between groups were performed by one-way analysis of variance followed by Dunn’s test for all pairwise comparison. *p*-values greater than 0.05 were deemed nonsignificant, and for significance, the indicated asterisks were applied: * *p* ≤ 0.05, ** *p* ≤ 0.01, *** *p* ≤ 0.001, and **** *p* ≤ 0.0001.

## 3. Results

### 3.1. Berberine Inhibits IgE Production in Human Tonsil Samples with No Effect on IgG Production or Cell Viability

**Figure 1:**
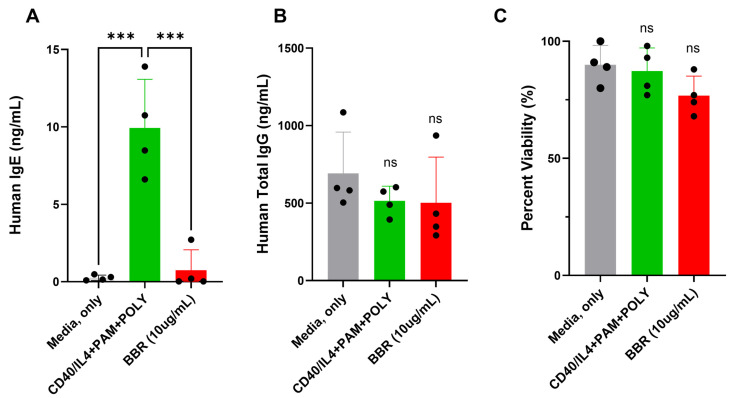
BBR reduces IgE without affecting the IgG of human tonsil samples with no effect on viability. Tonsil samples were unstimulated (media, only), stimulated (CD40/IL-4 + PAM + POLY), or stimulated and treated with 10 μg/mL of Berberine for 10 days. (**A**) IgE and (**B**) IgG production was quantified by ELISA. (**C**) Cell viability was determined by Trypan blue exclusion. BBR = Berberine. PAM = Pam3CSK4, POLY= Poly (I:C). N = 4, *** *p* ≤ 0.001, ns = not significant. Each dot represents a single biological replicate.

Human tonsil samples, which contain a mixed population of IgE- and IgG-producing plasma cells, were treated with BBR at 10 μg/mL. BBR treatment reduced the production of IgE (Figure 1A) without affecting the IgG production (Figure 1B) or cell viability (Figure 1C) of human tonsil cells. IgE+ plasma cells are a minor population in human samples, including tonsils, and IgE plasma cell isolation is difficult. Therefore, U266 and ARH-77 were used as surrogate plasma cells that produce IgE and IgG, respectively.

### 3.2. Berberine Inhibits IgE Production with No Effect on IgG Production in Myeloma Cell Lines

**Figure 2:**
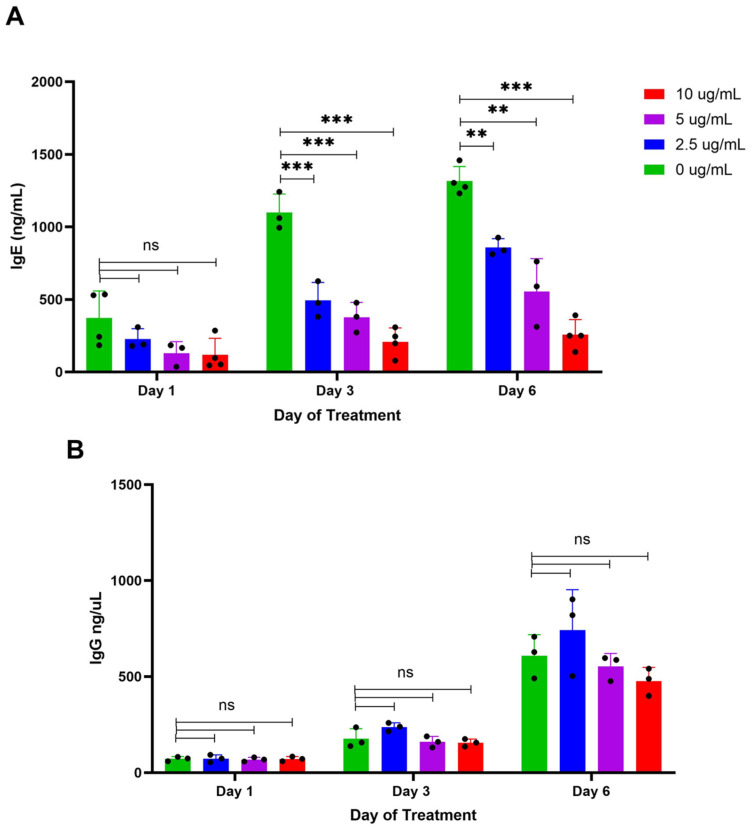
BBR decreases IgE secretion but not IgG secretion. The expression of IgE by IgE-producing myeloma cells, U266, was determined by ELISA following berberine (BBR) treatment at 10, 5, and 2.5 μg/mL compared to the equivalent concentration of DMSO (0 μg/mL) for 6 days (**A**). The expression of IgG by IgG-producing myeloma cells, ARH-77, was determined by ELISA, treated as U266 (**B**). N = 3–4, ** *p* ≤ 0.01, *** *p* ≤ 0.0001, ns = not significant. Each dot represents a single biological replicate.

To evaluate the specificity of BBR for IgE production over IgG production in vitro, U266 (IgE plasma cells) and ARH-77 (IgG plasma cells) were cultured at 2.5, 5, and 10 μg/mL for 6 days. On days 1, 3, and 6, supernatants were collected for assessment of IgE or IgG concentration with an ELISA, compared to the vehicle control, DMSO (0 μg/mL). BBR demonstrated dose-dependent effects on U266 IgE levels over the course of 6 days (Figure 2A) with no significant effect on IgG production by IgG-producing ARH-77 cells (Figure 2B). These results are consistent with the specificity of BBR on IgE production, in vitro.

### 3.3. The Reduction in IgE Production by Berberine Is Not Due to the Reduction in Cell Viability of IgE Plasma Cells

**Figure 3:**
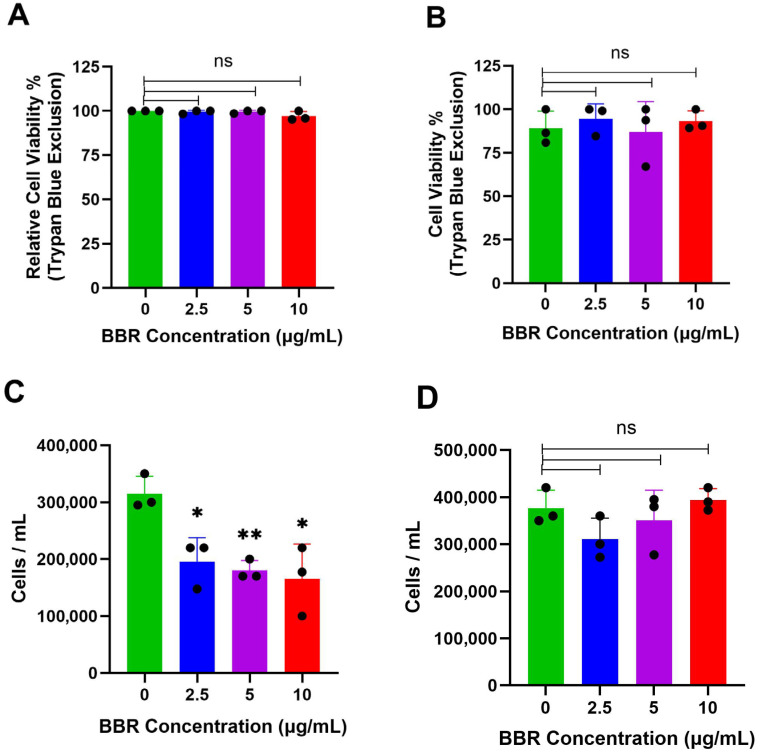
BBR does not affect the viability of IgE or IgG producing cells. (**A**) The viability of IgE by IgE-producing myeloma cells, U266, was determined by trypan blue exclusion assay, following berberine (BBR) treatment at 10, 5, and 2.5 μg/mL compared to the equivalent concentration of DMSO (0 μg/mL) for 6 days. (**B**) The same was performed for IgG-producing myeloma cells, ARH-77. (**C**) Total cell count determined by Trypan Blue was used to determine total cells per mL of U266 cells and (**D**) ARH-77 cells. N = 3. BBR= Berberine. * *p* ≤ 0.05, ** *p* ≤ 0.01, ns = not significant. Each dot represents a single biological replicate.

To evaluate if the observed reduction in IgE production was a result of cell death, cell viability was assessed by trypan blue exclusion. After 6 days of treatment, when compared to DMSO-treated controls, there was no reduction in the viability of IgE-producing cells (Figure 3A). Similarly, there were no effects on cell viability in IgG-producing ARH-77 cells, after 6 days of treatment, compared to DMSO-treated controls (Figure 3B). BBR treatment reduced the total cell number of U266, and this was not observed in ARH-77 cells (Figure 3C,D). Hence, the mechanism of IgE reduction is not due to cell death, but likely due to the halting of cell cycle progression, together with or independently of, the downregulation of IgE expression.

### 3.4. Berberine Treatment Reduced Proliferative Capacity of IgE Plasma Cells with Minimal Effect on IgG Plasma Cells

**Figure 4:**
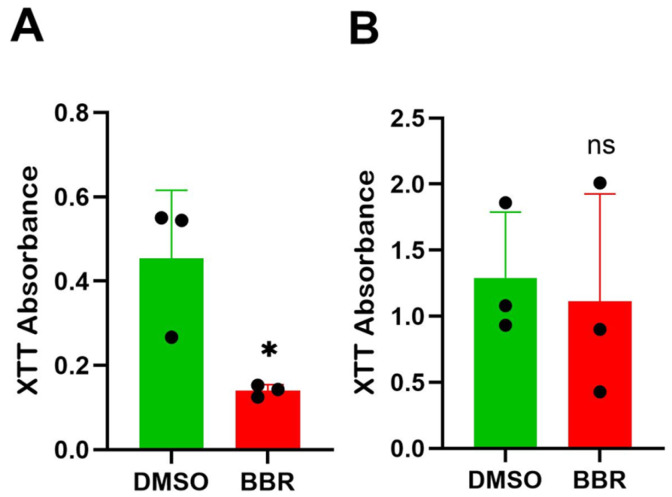
BBR affects proliferation of IgE-producing myeloma cells with no effect on IgG-producing myeloma cells. (**A**) The proliferation of IgE by IgE-producing myeloma cells, U266, was determined by XTT assay, following berberine (BBR) treatment at 10 μg/mL compared to the equivalent concentration of DMSO (0 μg/mL) for 6 days. (**B**) The same was performed for IgG-producing myeloma cells, ARH-77. N = 3. BBR = berberine, DMSO = dimethyl sulfoxide. * *p* ≤ 0.05, ns = not significant. Each dot represents a single biological replicate.

As indicated above, the reduction in IgE is not due to the BBR treatment induction of cell death. XTT assay demonstrated a halt in proliferation in IgE-producing U266 cells treated for 6 days at 10 μg/mL of BBR compared to the DMSO-treated controls (Figure 4A). In contrast, there was no significant difference between groups for ARH-77 cells (Figure 4B), suggesting that regulation by BBR occurs at the level of molecular regulation.

### 3.5. Berberine Treatment Alters Cell Cycle and p53 RNA Expression

**Figure 5:**
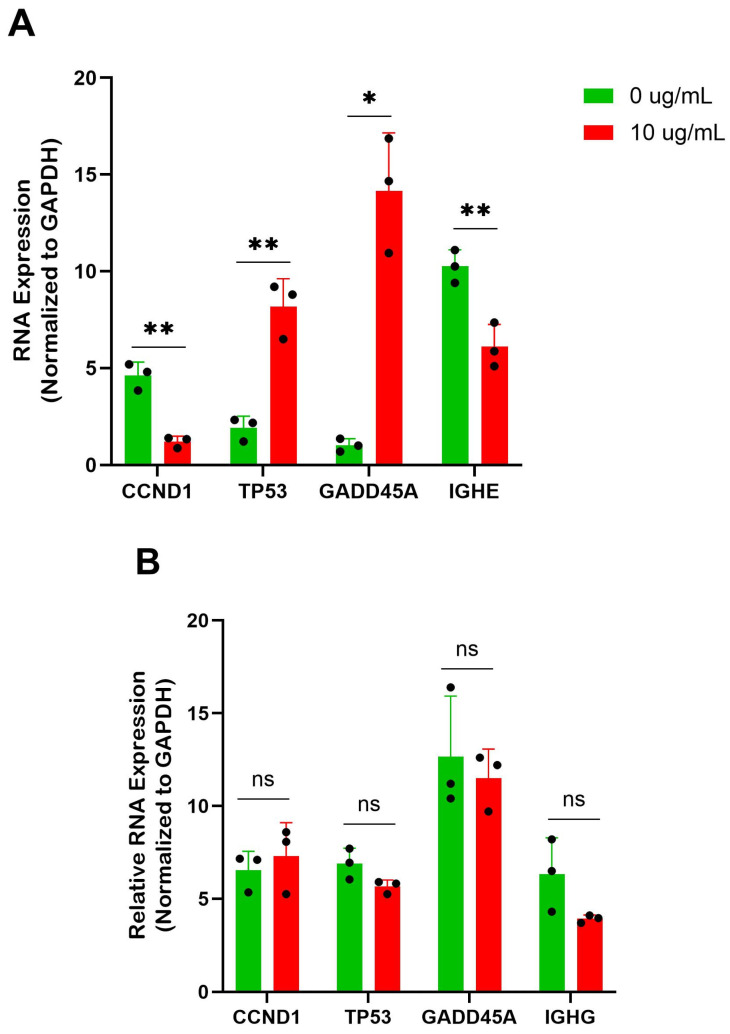
BBR affects cell cycle genes of IgE-producing myeloma cells with no effect on IgG-producing myeloma cells. (**A**) qRT-PCR of IgE heavy chain (*IGHE*) and cell cycle genes cyclin D1 (*CCND1*), tumor protein 53 (*TP53*), and growth arrest and DNA-damage inducible alpha (*GADD45A*), in IgE-producing U266 were performed after 6 days of berberine (BBR) treatment at 10 μg/mL compared to the equivalent concentration of DMSO (0 μg/mL). qPCR analysis of cell cycle proteins of IgE-producing U266 was performed after 6 days of berberine (BBR) treatment at 10 μg/mL compared to the equivalent concentration of DMSO (0 μg/mL) and data were normalized to glyceraldehyde-3-phosphate dehydrogenase (GAPDH) expression. (**B**) The same was performed for ARH-77, with analysis of IgG heavy chain (*IGHG*). N = 3. * *p* ≤ 0.05, ** *p* ≤ 0.01, ns = not significant. Each dot represents a single biological replicate.

Reduction in proliferative capacity without cell death implies alterations in the cell cycle. Previous work in our lab has demonstrated that small molecule natural compounds act to modulate IgE expression and pathways including cell cycle, p53, and DNA replication [11,12]. To confirm that there was a functional reduction in IgE heavy chain expression with no effect on IgG heavy chain transcription, qRT-PCR was performed. The expression of the IgE heavy chain (*IGHE*) was significantly decreased (Figure 5A); in contrast, IgG heavy chain (*IGHG*) expression was not significantly affected (Figure 5B).

Focusing on the p53 and cell cycle arrest pathways, we wanted to validate the expression of some notable genes by qRT-PCR. After 6 days of treatment at 10 μg/mL of BBR compared to the DMSO-treated controls, there was a significant increase in tumor protein 53 *(TP53*) and growth arrest and DNA-damage inducible alpha (*GADD45A*) expression and a significant reduction in cyclin D1 (*CCDN1*) in U266 cells at the RNA level (Figure 5A). This alteration was not seen in the BBR-treated ARH-77 cells (Figure 5B).

### 3.6. Berberine Treatment Alters Protein Expression of Cell Cycle and p53 Pathway

**Figure 6:**
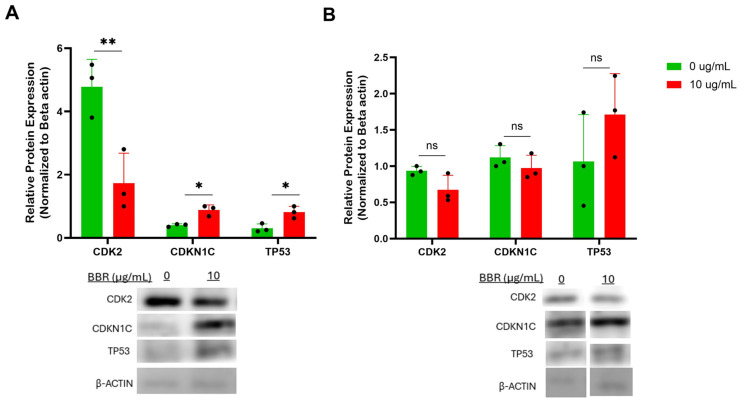
BBR affects cell cycle genes of IgE-producing myeloma cells with no effect on IgG-producing myeloma cells. (**A**) Western Blot analysis of cell cycle genes in IgE-producing U266 was performed after 6 days of berberine (BBR) treatment at 10 μg/mL compared to the equivalent concentration of DMSO (0 μg/mL). (**B**) The same was performed for ARH-77. N = 3. CDK2 = cyclin dependent protein kinase 2, CDKN1C = cyclin dependent kinase inhibitor 1C, TP53 = tumor protein 53. * *p* ≤ 0.05, ** *p* ≤ 0.01, ns = not significant. Each dot represents a single biological replicate.

Protein analysis was performed for the differential expression of markers of the p53 and cell cycle pathways. Western blot analysis of U266 cells after 6 days of BBR treatment at 10 μg/mL demonstrated a significant increase in protein expression of p53 and cyclin dependent kinase inhibitor (CDKN) 1C levels, and a significant decrease in cyclin dependent kinase 2 (CDK2) expression (Figure 6A). Again, there were no significant changes to any of these proteins in ARH-77 cells (Figure 6B). Together, the validated markers of cell cycle arrest and p53 activation may contribute to the mechanism of BBR suppression of IgE production, while not affecting the IgG production of IgG-producing cells.

### 3.7. Non-Coding RNA Regulation of Berberine Treatment in IgE Production

Regulation can occur by transcriptional, post-transcriptional and post-translational mechanisms. To further explore the specificity of BBR in IgE-producing plasma cells, without affecting IgG-producing plasma cells, we investigated miRNA expression. Non-Coding RNAs, including miRNAs, have distinct cell-type specific expression patterns, while also exhibiting developmental-stage specific expression patterns [13]. miRNAs have been connected to various allergic diseases, demonstrating differential expression and serving as diagnostic biomarkers and functioning as non-classical epigenetic regulators [14,15]. The ability to target specific miRNAs by BBR is an attractive avenue of exploration.

**Figure 7:**
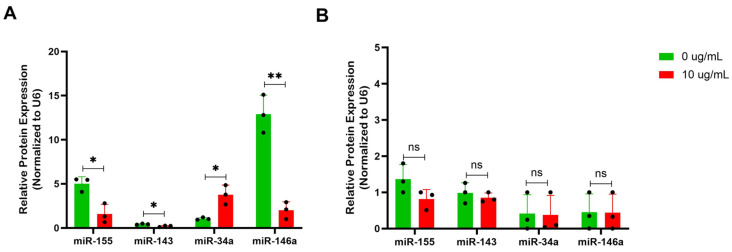
BBR affects expression of miRNAs involved in modulation of the p53 pathway of IgE-producing myeloma cells with no effect on IgG-producing myeloma cells. (**A**) miRNA isolation and qPCR analysis of miRNAs (miR) implicated IgE-regulation, miR-155, miR-143, miR-34a, and miR-146a were performed on U266 after 6 days of berberine (BBR) treatment at 10 μg/mL compared to the equivalent concentration of DMSO (0 μg/mL). (**B**) The same was performed for ARH-77. N = 3. * *p* ≤ 0.05, ** *p* ≤ 0.01, ns = not significant. Each dot represents a single biological replicate.

Investigation into miRNAs that regulate B cell or plasma cell development and the p53 and cell cycle pathways implicated miR-155, miR-143, miR-34a, and miR-146a [16,17,18,19,20,21,22,23]. Following treatment with 10 μg/mL of BBR, U266 cells demonstrated an increased expression in p53 activator miR-34a and reduction in p53 suppressors miR-155 and miR-146a (Figure 7A). The levels of these miRNAs were much lower in ARH-77 and not affected by BBR treatment (Figure 7B). miR-34a may be an important miRNA for BBR activity, serving as a link between GADD45A and p53 regulation.

## 4. Discussion

BBR demonstrated the ability to significantly reduce the IgE production of primary tonsil cell cultures and IgE-producing plasma cells U266 in vitro. However, there was no effect on IgG production by primary tonsil cells or IgG-producing plasma cells, ARH-77. The expression of the IgE heavy chain was significantly reduced by BBR treatment. Mechanistically, GADD45A, p53, and miR-34a upregulation highlights the selective targeting of BBR for IgE transcription and cell cycle regulation in IgE plasma cells. CCND1 and CDK2 inhibition and CDKN1C upregulation also validated cell cycle regulation by p53 [24]. Hence, BBR induces a controlled checkpoint response rather than cytotoxicity that preserves immunocompetence by not disturbing IgG responses. This is of great benefit over current biologics, which neutralize IgE already in circulation [25], while BBR prevents IgE production upstream. BBR offers an attractive alternative or adjunctive therapy at a potentially lower cost.

IgE production requires class-switch recombination, which is regulated at the transcriptional level of the constant heavy-chain genes, and our data demonstrate that BBR suppresses IgE expression without cytotoxicity. Activation of nuclear factor kappa B (NFκB) signaling pathways is one of the upstream mechanisms underlying B-cell activation and germline transcription of IgE. It has been demonstrated in in vivo allergic rhinitis models that BBR treatment targets were primarily involved in pathways such as NFκB, IL-17, tumor necrosis factor, and inflammatory responses [26]. Previous work in our lab demonstrated that in peripheral blood mononuclear cells, BBR treatment significantly suppressed the increase in p-IκBꭤ levels upon stimulation compared with the untreated cells. Moreover, expressions of signal transducer and activator of transcription (STAT)-3 and T-bet were markedly elevated, while Foxp3 expression displayed an upward trend. Similarly, IL-10 and interferon-ϒ were modestly upregulated, accompanied by a pronounced reduction in IL-5 expression [5]. Our lab has also shown that downregulation of IgE may be linked to inhibition of XBP1, BLIMP1, STAT6 and mitochondrial metabolic activity, together with enhanced B cell lymphoma 6 (BCL-6), leading to restricted energetic support and transcriptional activation for IgE heavy chain and light chain synthesis and antibody glycosylation in U266 IgE myeloma cells [4]. In human tonsil cells, BBR also modulated STAT6, NFκB1, NFκB1A, and BCL-6 expression, while inhibiting STAT6 binding activity to the IgE promoter [27]. This finding suggests that the reduction in IgE synthesis by BBR is not due to overall suppression of gene transcription, but instead involves multiple, specific regulatory pathways, providing support for BBR use as a therapeutic option to provide long-term and sustained effectiveness.

Our observation that BBR can differentially regulate IgE and IgG production in vitro confirms and extends data obtained in previous studies using mouse models [27]. The IgE-producing plasma cells may be unique to other B cell isotypes and can be exploited for targeting IgE-mediated allergic disease. To understand the mechanism by which BBR would be able to specifically target IgE production, without affecting IgG and consequently the entire immune system, its effect on miRNA expression was investigated. miRNAs are non-coding RNAs that are small, around 22 nucleotides in length, single-stranded, transcripts that work through the regulation of target gene expression, including transcriptional and post-transcriptional regulation of genes involved in nearly all biological processes [28]. Importantly, miRNAs are cell-specific, even to the level of stage of development. It is probable that miRNAs of IgE-producing plasma cells would be different from IgG-producing plasma cells. However, there has not been an unbiased comparison of sorted isotypes of B cells assessing miRNA signatures by sequencing.

Other natural compounds have shown modulation of miRNA expression including resveratrol, curcumin, genistein, epigallocatechin-3-gallate, and quercetin [29,30,31,32,33,34,35]. There are different ways that compounds can affect miRNA expression levels: (1) direct binding to the miRNA sequence, (2) modulate transcription factors that bind miRNA promoters, (3) influence enzymes involved in miRNA maturation, for example, Dicer and Drosha, (4) DNA methylation or other epigenetic changes that alter miRNA accessibility, and (5) modulation of lncRNA or other competitive endogenous RNAs that can competitively bind sites of the target miRNA. BBR has previously demonstrated modulations to miR-21 of U266 and RPMI-8266 cells, another IgG-producing myeloma cell line, potentially through IL-6/STAT3, and the consequential increase in programmed cell death 4 gene expression [36].

miR-34a is regulated by p53 due to the p53-binding site located in its promoter, whereby upon p53 binding, a positive feedback mechanism ensues and results in the increase in p53 activity [37,38]. The reduction in sirtuin 1 (SIRT1) expression accounts for this activity, as when activated, SIRT1 deacetylates p53, inhibiting its transcriptional activation, and reducing CDKN1A expression and promoting apoptosis, G2 arrest, and senescence [37]. Similarly, increased miR-34a expression has demonstrated anticancer activity in a similar mechanism [29]. miR-34a has also demonstrated the ability to bind to double minute 4 (MDM4), a negative regulator of TP53. Knockdown of MDM4 led to activation of p53 and induced cell-cycle arrest in Burkitt lymphoma cell lines [39]. Interestingly, knockdown of MDM4 displayed increased recruitment of p53 to promoters of its proapoptotic targets in human melanoma cells [40]. In synergy, miR-155 negatively regulates p53, particularly in the context of B lymphocytes, whereby high levels of miR-155 decrease p53 activity, and hence the development of B cell cancer through enhanced proliferation [41,42]. miR-146a plays a crucial role in B cell activation and differentiation, through regulation of target genes acting to negatively regulate B cell function. miR-146a attenuates apoptosis through targeting of the p53 pathway [43].

Interestingly, miR-34a has previously been implicated in GADD45A regulation in osteosarcoma in vitro through antagonism of Dicer-antisense 1, which also modulates p53 expression [44]. The elucidation of the role of GADD45A in plasma cells has been demonstrated previously, with expression being downregulated in IgE-producing plasma cells and plasmablasts compared to those of the IgG isotype [9]. The ability of BBR to increase its expression is of great significance. GADD45A-mediated DNA demethylation allows for specific epigenetic activation of genes of cellular responses to stress or differentiation [45,46]. GADD45A also suppresses STAT3 transcription and may trigger AMP-activated protein kinase, which regulates metabolism, and serves as a transcriptional coregulator of various nuclear receptors [45]. GADD45A is an attractive target for the seen BBR-induced effects.

It has been demonstrated through microarray analysis of murine mast cells with atopic dermatitis that BBR inhibited antigen-stimulated increased the expression of mucosa-associated tissue lymphoma translocation protein-1 (MALT1) and eukaryotic translation initiation factor 3 [47]. The authors pose that their downregulation inhibits MIF (macrophage migration inhibitory factor) and IL-4 expression, and hence the observed reduction in serum IgE levels. MALT1 plays a crucial role in the activation of the NFκB pathway, with implications in lymphoid malignancies including B cell lymphomas. Deficiencies in MALT lead to impaired B cell responses including IgE levels and altered B cell development. MALT1-deficient mice demonstrated impaired IgE production, suggesting their role in B cell class switching to IgE-producing plasma cells. Interestingly, MALT1 has been demonstrated to inhibit p53-mediated apoptosis whereby p53 can also downregulate MALT1 expression. In lymphomas, MALT1 promotes cancer development by activating NFκB and inhibiting p53-mediated apoptosis. In all, these studies suggest the value of BBR for food allergies and other mast cell and IgE-mediated disorders.

Limitations to this study include the lack of in vivo models supporting the proposed mechanism. Murine allergy models treated with BBR and subsequent isolation of immune cells for expression of these markers would confirm the BBR-specific effects. Tonsil and plasma cell lines lack the heterogeneity of IgE responses that would otherwise be seen in vivo and in patients.

Future work aims to target miR-34a and transcription factor GADD45A with gene editing technology to confirm that their modulation is imperative to the function of BBR in IgE-producing cells. Additionally, further characterization of the effect of GADD45A through cell cycle, apoptosis and senescence assays are crucial. Research also suggests that circulating miRNA expression levels may be strongly influenced by diet [48] so it may be beneficial to determine the expression of these miRNAs depending on food allergy type and if that influences the efficacy of BBR. The expression of p53 has demonstrated physiological significance of pathological IgE-mediated stimulation, with its reduction enhancing anaphylaxis in vivo [49]. As p53 also plays a central role in the regulation of cell cycle arrest, apoptosis, and senescence, its modulation by BBR may also extend into various cancers. While IgG myeloma cells were not affected as the IgE myeloma cells were at the same concentration, a higher concentration may be investigated for the inhibition of both cell types.

Mechanistically, BBR effects on miRNA expression and transcription factors unique to IgE B cells may explain this IgE-specific effect. Gene expression changes that suppress IgE without affecting IgG allow for the lack of overall immune suppression with BBR treatment. Therefore, BBR has the potential to be a novel and accessible therapy applied to other IgE-mediated allergic diseases including allergic asthma, chronic urticaria, and atopic dermatitis.

## 5. Conclusions

**Figure 8:**
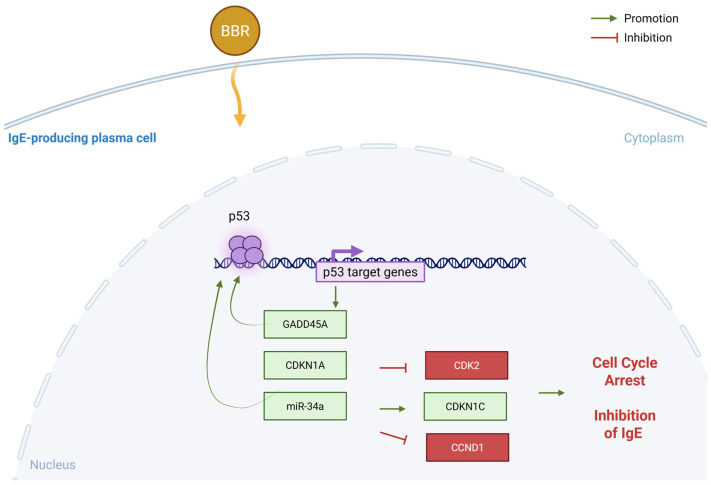
Proposed mechanism—BBR activates GADD45A through modulation of miR-34a specifically in IgE-producing myeloma cells. Berberine treatment results in p53 activation specific to IgE-producing cells that results in GADD45A and CDKN1A upregulation and consequently inhibiting cell cycle regulator CDK2 and CCND1. This specificity could be due to non-coding regulation by miRNA-34a, specific to IgE producing cells. Figure made in BioRender. BBR = berberine, CDK2 = cyclin dependent kinase 2, CDKN = cyclin dependent kinase inhibitor, GADD45A = growth arrest and DNA damage inducible, alpha, miR = miRNA, p53 = tumor protein 53. Yellow arrow indicates uptake of BBR into the cell.

IgE+ plasma cells are a minor population of the tonsils, directing us to use U266 and ARH-77 plasma cell lines as surrogate cells for evaluating the IgE-specific regulation of BBR. Mechanistically, BBR may preferentially target IgE-producing cells over IgG-producing cells due to their cell-specific expression of regulatory miRNAs and transcription factors that directly increase GADD45A expression and consequently induce cell cycle arrest (Figure 8).

## Data Availability

The original contributions presented in this study are included in the article. Further inquiries can be directed to the corresponding author.

## Supplementary Materials

The following supporting information can be downloaded at: https://www.mdpi.com/article/10.3390/cells14221799/s1. Table S1: Primer pairs used in this study.

## Abbreviations

The following abbreviations are used in this manuscript:

BBR	Berberine
BCL-6	B cell lymphoma 6
CCND1	Cyclin D1
CDK2	Cyclin dependent kinase 2
CDKN	Cyclin dependent kinase inhibitor
DMSO	Dimethyl sulfoxide
FAHF	Food allergy herbal formula
FBS	Fetal bovine serum
GADD45A	Growth arrest and DNA damage inducible, alpha
GAPDH	Glyceraldehyde-3-phosphate dehydrogenase
Ig	Immunoglobulin
IL	Interleukin
lncRNA	Long non-coding RNA
MALT1	Mucosa-associated tissue lymphoma translocation protein-1
MDM4	Mouse double minute 4
miRNA	MicroRNA
ncRNA	Non-coding RNA
NFκB	Nuclear factor kappa B subunit
qRT-PCR	Quantitative reverse transcriptase polymerase chain reaction
RIPA	Radioimmunoprecipitation assay
RPMI	Roswell Park Memorial Institute
SIRT1	Sirtuin 1
STAT	Signal transducer and activator of transcription
TP53	Tumor protein 53
XTT	2,3-Bis-(2-Methoxy-4-Nitro-5-Sulfophenyl)-2H-Tetrazolium-5-Carboxanilide

## References

[1] U.S. Department of Agriculture. Food Allergies: The “Big 9”. https://www.fsis.usda.gov/food-safety/safe-food-handling-and-preparation/food-safety-basics/food-allergies-big-9.

[2] Network A.A. A Complete Guide to Allergies. https://allergyasthmanetwork.org/allergies/.

[3] Cildag S., Senturk T. (2018). The effect of omalizumab treatment on IgE and other immunoglobulin levels in patients with chronic spontaneous urticaria and its association with treatment response. Postepy Dermatol. Alergol..

[4] Yang N., Maskey A.R., Srivastava K.D., Kim M., Wang Z., Musa I., Shi Y., Gong Y., Fidan O., Wang J. (2023). Inhibition of pathologic immunoglobulin E in food allergy by EBF-2 and active compound berberine associated with immunometabolism regulation. Front. Immunol..

[5] Yang N., Wang J., Liu C., Song Y., Zhang S., Zi J., Zhan J., Masilamani M., Cox A., Nowak-Wegrzyn A. (2014). Berberine and limonin suppress IgE production by human B cells and peripheral blood mononuclear cells from food-allergic patients. Ann. Allergy Asthma Immunol..

[6] Srivastava K.D., Cao M., Fidan O., Shi Y., Yang N., Nowak-Wegrzyn A., Miao M., Zhan J., Sampson H.A., Li X.M. (2023). Berberine-containing natural-medicine with boiled peanut-OIT induces sustained peanut-tolerance associated with distinct microbiota signature. Front. Immunol..

[7] Buell D.N., Fahey J.L. (1969). Limited periods of gene expression in immunoglobulin-synthesizing cells. Science.

[8] Zhang Y., Fear D.J., Willis-Owen S.A., Cookson W.O., Moffatt M.F. (2016). Global gene regulation during activation of immunoglobulin class switching in human B cells. Sci. Rep..

[9] Ramadani F., Bowen H., Gould H.J., Fear D.J. (2019). Transcriptional Analysis of the Human IgE-Expressing Plasma Cell Differentiation Pathway. Front. Immunol..

[10] Panganiban R.P., Pinkerton M.H., Maru S.Y., Jefferson S.J., Roff A.N., Ishmael F.T. (2012). Differential microRNA epression in asthma and the role of miR-1248 in regulation of IL-5. Am. J. Clin. Exp. Immunol..

[11] Yang N., Srivastava K.D., Chen Y., Li H., Maskey A.R., Yoo P., Liu X., Tiwari R.K., Geliebter J., Nowak-Wegrzyn A. (2024). Sustained silencing peanut allergy by xanthopurpurin is associated with suppression of peripheral and bone marrow IgE-producing B cell. Front. Immunol..

[12] Musa I., Wang Z., Yang N., Li X. (2024). Formononetin inhibits IgE by huPlasma/PBMCs and mast cell/basophil activation via JAK/STAT/PI3-Akt pathways. Front. Immunol..

[13] Malumbres R., Sarosiek K.A., Cubedo E., Ruiz J.W., Jiang X., Gascoyne R.D., Tibshirani R., Lossos I.S. (2008). Differentiation stage–specific expression of microRNAs in B lymphocytes and diffuse large B-cell lymphomas. Blood.

[14] Alashkar Alhamwe B., Potaczek D.P., Miethe S., Alhamdan F., Hintz L., Magomedov A., Garn H. (2021). Extracellular Vesicles and Asthma-More Than Just a Co-Existence. Int. J. Mol. Sci..

[15] Grueso-Navarro E., Navarro P., Laserna-Mendieta E.J., Lucendo A.J., Arias-González L. (2023). Blood-Based Biomarkers for Eosinophilic Esophagitis and Concomitant Atopic Diseases: A Look into the Potential of Extracellular Vesicles. Int. J. Mol. Sci..

[16] Okada N., Lin C.-P., Riberio M.C., Biton A., Lai G., He X., Bu P., Vogel H., Jablons D.M., Keller A.D. (2014). A positive feedback between p53 and miR-34 miRNAs mediates tumor suppression. Genes Dev..

[17] Nguyen D.C., Duan M., Ali M., Ley A., Sanz I., Lee F.E. (2021). Plasma cell survival: The intrinsic drivers, migratory signals, and extrinsic regulators. Immunol. Rev..

[18] Navarro F., Lieberman J. (2015). miR-34 and p53: New Insights into a Complex Functional Relationship. PLoS ONE.

[19] Vigorito E., Perks K.L., Abreu-Goodger C., Bunting S., Xiang Z., Kohlhaas S., Das P.P., Miska E.A., Rodriguez A., Bradley A. (2007). microRNA-155 regulates the generation of immunoglobulin class-switched plasma cells. Immunity.

[20] Basso K., Schneider C., Shen Q., Holmes A.B., Setty M., Leslie C., Dalla-Favera R. (2012). BCL6 positively regulates AID and germinal center gene expression via repression of miR-155. J. Exp. Med..

[21] Lu D., Nakagawa R., Lazzaro S., Staudacher P., Abreu-Goodger C., Henley T., Boiani S., Leyland R., Galloway A., Andrews S. (2014). The miR-155-PU.1 axis acts on Pax5 to enable efficient terminal B cell differentiation. J. Exp. Med..

[22] Borbet T.C., Hines M.J., Koralov S.B. (2021). MicroRNA regulation of B cell receptor signaling. Immunol. Rev..

[23] Guo Q., Zhang J., Li J., Zou L., Zhang J., Xie Z., Fu X., Jiang S., Chen G., Jia Q. (2013). Forced miR-146a expression causes autoimmune lymphoproliferative syndrome in mice via downregulation of Fas in germinal center B cells. Blood.

[24] Hernández Borrero L.J., El-Deiry W.S. (2021). Tumor suppressor p53: Biology, signaling pathways, and therapeutic targeting. Biochim. Biophys. Acta Rev. Cancer.

[25] Villamañán E., Laorden D., Granda P., Sobrino C., De Andrés S., Carpio C., Domínguez-Ortega J., Romero D., Mariscal P., De Las Vecillas L. (2024). Current Biologic Therapies for Severe Asthma and Real-World Data: Are Expectations Being Met?. J. Clin. Med..

[26] Luo G., Gao M., Lin Q. (2024). Integration of bioinformatics analysis, molecular docking and animal experiments to study the therapeutic mechanisms of berberine against allergic rhinitis. Sci. Rep..

[27] Maskey A.R., Carnazza M., Spears M., Hemmindinger S., Kopulos D., Yang N., Islam H.K., Moscatello A.L., Geliebter J., Tiwari R.K. (2025). Berberine Suppression of Human IgE but Not IgG Production via Inhibition of STAT6 Binding Activity at IgE Promoter by BCL6. Cells.

[28] O’Brien J., Hayder H., Zayed Y., Peng C. (2018). Overview of MicroRNA Biogenesis, Mechanisms of Actions, and Circulation. Front. Endocrinol..

[29] Alnugaydan A.M. (2020). Targeting micro-RNAs by natural products: A novel future therapeutic strategy to combat cancer. Am. J. Transl. Res..

[30] Yarahmadi S., Sotoudeheian M., Farahmandian N., Mohammadi Y., Koushki M., Babaeenezhad E., Yousefi Z., Fallah S. (2025). Effect of resveratrol on key signaling pathways including SIRT1/AMPK/Smad3/TGF-β and miRNA-141 related to NAFLD in an animal model. Res. Pharm. Sci..

[31] Chen J., Xiong D., Long M. (2025). Curcumin Attenuates Fumonisin B1-Induced PK-15 Cell Apoptosis by Upregulating miR-1249 Expression to Inhibit the IRE1/MKK7/JNK/CASPASE3 Signaling Pathway. Antioxidants.

[32] Qin H., Song Z., Zhao C., Li S., Ali A., Zheng W. (2023). miR-363-3p/PTEN is involved in the regulation of lipid metabolism by genistein in HepG2 cells via ERβ. Phytomedicine.

[33] Yang X., Jiang W., Kong X., Zhou X., Zhu D., Kong L. (2022). Genistein Restricts the Epithelial Mesenchymal Transformation (EMT) and Stemness of Hepatocellular Carcinoma via Upregulating miR-1275 to Inhibit the EIF5A2/PI3K/Akt Pathway. Biology.

[34] Dharshini L.C.P., Mandal A.K.A. (2024). Regulation of gene expression by modulating microRNAs through Epigallocatechin-3-gallate in cancer. Mol. Biol. Rep..

[35] Ramos Y.A.L., Souza O.F., Novo M.C.T., Guimarães C.F.C., Popi A.F. (2021). Quercetin shortened survival of radio-resistant B-1 cells in vitro and in vivo by restoring miR15a/16 expression. Oncotarget.

[36] Lao X., Gu J., Zhu R., Feng M., Zhu X., Li Y., Fei J. (2014). Integrative analysis of differential miRNA and functional study of miR-21 by seed-targeting inhibition in multiple myeloma cells in response to berberine. BMC Syst. Biol..

[37] Feliciano A., Sánchez-Sendra B., Kondoh H., Lleonart M.E. (2011). MicroRNAs Regulate Key Effector Pathways of Senescence. J. Aging Res..

[38] Capaccia C., Diverio S., Zampini D., Guelfi G. (2022). The Complex Interaction Between P53 and miRNAs Joins New Awareness in Physiological Stress Responses. Cells.

[39] Hüllein J., Słabicki M., Rosolowski M., Jethwa A., Habringer S., Tomska K., Kurilov R., Lu J., Scheinost S., Wagener R. (2019). MDM4 Is Targeted by 1q Gain and Drives Disease in Burkitt Lymphoma. Cancer Res..

[40] Gembarska A., Luciani F., Fedele C., Russel E., Dewaea M., Villar S., Zwolinska A., Haupt S., de Lange J., Yip D. (2012). MDM4 is a key therapeutic target in cutaneous melanoma. Nat. Med..

[41] Cui B., Chen L., Zhang S., Mraz M., Fecteau J., Yu J., Ghia E., Zhang L., Bao L., Rassenti L. (2014). MicroRNA-155 influences B-cell receptor signaling and associates with aggressive disease in chronic lymphocytic leukemia. Blood.

[42] Bouamar H., Jiang D., Wang L., Lin A., Ortega M., Aguiar R. (2015). MicroRNA 155 control of p53 activity is context dependent and mediated by Aicda and Socs1. Mol. Cell. Biol..

[43] Pan J., Tang Y., Yu J., Zhang J., Wang C., Gu J. (2009). miR-146a attenuates apoptosis and modulates autophagy by targeting TAF9b/P53 pathway in doxorubicin-induced cardiotoxicity. Cell Death Dis..

[44] Wang F., Kong L., Pu Y., Chao F., Zang C., Qin W., Zhao F., Cai S. (2021). Long Noncoding RNA DICER1-AS1 Functions in Methylation Regulation on the Multi-Drugresistance of Osteosarcoma Cells via miR-34a-5p and GADD45A. Front. Oncol..

[45] Palomer X., Salvador J., Grinan-Ferre C., Barroso E., Pallas M., Vazquez-Carrera M. (2023). GADD45A: With or without you. Med. Res. Rev..

[46] Barreto G., Schafer A., Marhold J., Stach D., Swaminathan S., Handa V., Doderlein G., Maltry N., Wu W., Lyko F. (2007). Gadd45a promotes epigenetic gene activation by repair-mediated DNA demethylation. Nature.

[47] Andoh T., Yoshihisa Y., Rehman M.U., Tabuchi Y., Shimizu T. (2021). Berberine induces anti-atopic dermatitis effects through the downregulation of cutaneous EIF3F and MALT1 in NC/Nga mice with atopy-like dermatitis. Biochem. Pharmacol..

[48] Ferrero G., Carpi S., Polini B., Pardini B., Nieri P., Impeduglia A., Grioni S., Tarallo S., Naccarati A. (2021). Intake of Natural Compounds and Circulating microRNA Expression Levels: Their Relationship Investigated in Healthy Subjects with Different Dietary Habits. Front. Pharmacol..

[49] Suzuki K., Murphy S.H., Xia Y., Yokota M., Nakagomi D., Liu F., Verma I.M., Nakajima H. (2011). Tumor suppressor p53 functions as a negative regulator in IgE-mediated mast cell activation. PLoS ONE.

