# Supplementary material for: A Small Molecule Compound, Berberine Reduces IgE but Not IgG Production via Promoting miRNA-34a-p53 Axis
Source: Cells. 2025 Nov 17;14(22):1799. doi: 10.3390/cells14221799 (PMC12650823; doi:10.3390/cells14221799)
Supplement: Supplementary file 1 [file cells-14-01799-s001.zip › cells-3939018-supplementary.pdf]

Table S1: Primer pairs used in this study.

Gene	Forward (5'-3')	Reverse (5'-3')
CCND1	ATGTTCGTGGCCTCTAAGATGA	CAGGTTCCACTTGAGCTTGTTC
TP53	TGCGTGTGGAGTATTTGGATG	TGGTACAGTCAGAGCCAACCTC
GADD45A	TCTGTTGCGAGAACGACATC	TCCCGGCAAAACAAATAAG
IGHE	ACCCTGGTCACCGTCTCCTCAG	CAGAGTCACGGAGGTGGCATT
IGHG	ACCCTGGTCACCGTCTCCTCAG	GTTCCACGACACCGTCACC
GAPDH	GGGTGTGAACCATGAGAAGTAT	GACTGTGGTCATGAGTCCTTC
miR-155	GTGCTGCAAACCAGGAAGG	-
miR-143	GTGCAGTGCTCATCTCGGT	-
miR-34a	TGGCAGTGTCTTAGCTGGTTGT	-
miR-146a	TTAATGCTAATGTGTAGGGGTT	-

CCND1= cyclin D1, GADD45A = growth arrest and DNA damage inducible, alpha, GAPDH = glyceraldehyde-3-phosphate dehydrogenase, IGH= Immunoglobulin heavy chain, miR = miRNA, TP53 = tumor protein 53
